# Cough—Experimental Study

**Published:** 1874-12

**Authors:** O. Kohts

**Affiliations:** Strasbourg


Cough—Experimental Study.—*By Dr. O. Kohts, of Strasbourg.* Irritation of the centripetal fibres of the vagus nerve produces reflex cough, and the experiments show it to lead from—

I. The pharyngeal nerve, the superior laryngeal and the vagus.

II. From the peripheral distribution of the vagus nerve. Cough appears—

*a.* On irritation of the mucous membrane of the pharynx, of the larynx (the fossa inter arytaenoidea, the plica glosso-epiglotica and the plica ary-epiglottica), the trachea, the point of bifurication and the bronchi;

*b.* On irritation of the costal pleura;

*c.* On irritation of the œsophagus. The experimental proofs of a so-called stomach cough are absent.

III. There is a central cough, which may be excited by direct irritation of the medulla oblongata.—*The Clinic, Oct.* 31, 1874.

